# The impact of the COVID-19 pandemic on osteoporotic fractures: a systematic review and meta-analysis

**DOI:** 10.1080/07853890.2025.2604391

**Published:** 2025-12-22

**Authors:** Peiyuan Tang, Ruijie Xiao, Wenfeng Xiao, Ting Wen, Yusheng Li, Bangbao Lu, Xucheng Yang

**Affiliations:** a Department of Orthopedics, Xiangya Hospital, Central South University, Changsha, Hunan, China; b National Clinical Research Center for Geriatric Disorders, Xiangya Hospital, Central South University, Changsha, China; c Xiangya School of Medicine, Central South University, Changsha, China

**Keywords:** SARS-CoV-2, COVID-19, osteoporosis, fracture, meta-analysis

## Abstract

**Background:**

Recent reports suggest that the COVID-19 pandemic and associated lockdowns may have influenced the epidemiology of osteoporotic fractures, but results vary across regions and fracture types. The aim of this study was to provide evidence-based insights into the impact of the pandemic on osteoporotic fracture incidence.

**Methods:**

We searched four databases (PubMed, Embase, Cochrane Library, and Web of Science) up to August 2025 for observational or retrospective studies comparing osteoporotic fracture incidence during the COVID-19 pandemic (2020) with the pre-pandemic period (2019). The primary outcome of interest was the change in fracture incidence, analysed using risk ratios (RR) with 95% confidence intervals (CI) in Review Manager 5.4. Subgroup analyses were performed by sex, geographic region, and fracture type.

**Results:**

Nine studies meeting the inclusion criteria were analysed. Overall, “all types” of osteoporotic fractures showed a significant decrease during the pandemic (RR = 0.85, 95% CI 0.80–0.91, *p* < 0.0001). Specifically, forearm fractures decreased significantly (RR = 0.87, 95% CI 0.79–0.96, *p* = 0.002). However, for the most clinically significant fractures, no statistically significant global change was found for hip fractures (RR = 0.93, 95% CI 0.76–1.15, *p* = 0.14) or vertebral fractures (RR = 1.35, 95% CI 0.85–2.15, *p* = 0.20). In regional subgroup analysis, hip fracture incidence decreased significantly in South America (RR = 0.79, *p* = 0.0004) and in both males and females, but no significant change was observed in Europe (RR = 0.92, 95% CI 0.81–1.04, *p* = 0.17).

**Conclusion:**

During the COVID-19 pandemic, there was a decrease in the incidence of minor fractures, such as those of the forearm, likely due to reduced outdoor activity. However, the incidence of major osteoporotic fractures (hip and vertebral) remained stable globally, with significant reductions observed only in specific regions like South America.

## 1. Introduction

COVID-19 is a disease caused by Severe Acute Respiratory Syndrome Coronavirus 2 (SARS-CoV-2), characterised by fever and respiratory symptoms, as well as fatigue, muscle aches, dizziness, and confusion [1,2]. COVID-19 was classified as a pandemic in March 2020 by the World Health Organisation (WHO) as a pandemic [3,4], and it primarily affects the elderly population due to their higher levels of frailty and associated comorbidities. Globally, more than half of the deaths from COVID-19 are in the elderly [5,6]. The severe form of this disease is associated with activation of the immune system, accompanied by an increase in the production of pro-inflammatory cytokines and an increase in C-reactive protein (CRP) levels [7,8]. Under the guidance of the WHO, many countries implemented lockdowns to control the spread of the disease [9]. However, this has also led to a reduced opportunity for patients to access healthcare services, particularly for older people who face difficulties seeking medical attention due to social isolation and restricted transportation. Indeed, changes in the lifestyle habits of older adults are likely to modify the risk factors associated with osteoporotic fractures. On the one hand, because of lockdowns during pandemics, older adults spend less time outdoors, reducing the likelihood of exposure to environmental travel hazards [6]. On the other hand, COVID-19 and its associated symptoms of fatigue, dizziness and confusion may in turn increase the risk of causing falls and fractures in older adults [10].

Osteoporosis is a common disease in older people [11]. It is a systemic disease characterised by progressive changes in both the quantity and quality of bone mass, which increases the risk of fractures even without trauma, and its incidence increases with age [12]. Osteoporotic fractures are a major public health and healthcare issue worldwide, as they are associated with high healthcare costs, occurrence rates, and mortality [13]. According to the International Osteoporosis Foundation, in people aged 50 and over, one in every three women and one in every five men will experience an osteoporotic fracture in their lifetime [14]. Worldwide, osteoporosis is estimated to cause 8.9 million fractures per year. Among these, hip, forearm, and vertebral fractures are the most common sites of fracture [13]. Kirwan et al. found that the blockade during COVID-19 led to a reduction in physical activity, causing sarcopenia and loss of muscle mass in older adults in particular [15]; Tang et al. [16] showed that prolonged immobilisation also increased the chances of falls in older adults caused by COVID-19-related factors, as well as chronic inflammation and frailty. Imaicela et al. found a steady increase in the incidence of hip fractures in Ecuador [17]. However, a study by Ojeda-Thies et al. showed a reduction of about a quarter of the brittle hip fracture cases admitted to their hospital during the blockade in Madrid, Spain, compared to the control period in 2018 and 2019 [6]. In Brazil and Chile, it also reported a decrease in the standardised incidence of hip fractures during the pandemic [17]. It is evident that the impact of the COVID-19 pandemic on osteoporotic fractures remains controversial, and we conducted a systematic review of the relevant literature to compare the incidence of osteoporotic fractures during the same period in 2019 and 2020 in order to assess the impact of the pandemic on osteoporotic fractures, to provide more evidence-based recommendations for the prevention of osteoporotic fractures, and to provide in-depth research on the post-new coronary infections of the skeletal muscle sequelae and evidence for the allocation of hospital orthopedic resources.

## 2. Methods

This meta-analysis was registered on the International Prospective Register of Systematic Reviews (ID: CRD42023425863) and carried out in accordance with methodological guidelines from the Cochrane Handbook for Systematic Reviews [18]. The findings were reported in accordance with the PRISMA (Preferred Reporting Items for Systematic Review and Meta-Analyses) statement (Supplementary Material S1) [19,20].

### 2.1. Search strategy

Four databases including Embase, PubMed/MEDLINE, Web of Science, and Cochrane Library were searched up to August 2025. The literature retrieval was conducted using a combination of subject terms and free words. The English search terms included Osteoporosis, Post Traumatic Osteoporosis, Senile SARS-CoV-2, 2019 Novel Coronavirus, COVID-19 Virus, etc (Supplementary Material S2).

### 2.2. Eligibility criteria

This system overview and meta-analysis follow the inclusion criteria of PICOs standard [21]. We included observational studies (including prospective or retrospective cohort studies) that assessed osteoporotic fractures during the COVID-19 pandemic in 2020 and during 2019 (pre-pandemic). Studies were excluded if they were narrative literature reviews (although their reference lists were explored for potentially eligible studies), and reports lacking a historical control group from the corresponding period in 2019 (pre-pandemic) (Supplementary Material S3).

### 2.3. Data extraction and quality assessment

Data extraction and quality assessment were independently performed by two reviewers (P.T. and T.W.), and discrepancies were solved by consensus with a third reviewer (Y.L.). The following data were extracted from the eligible studies: authors, whether there was a lockdown in the country of study, fracture type, outcome measures, study design, country, age, and level of evidence. The primary outcome measure was the change in the incidence of osteoporotic fractures comparing the pandemic period (2020) to the pre-pandemic period (2019). The quality of included cohort and case studies was assessed using the Newcastle–Ottawa Scale (NOS) [22].

### 2.4. Subgroup analysis

We performed three subgroup analyses. The first subgroup was based on sex, the second subgroup was based on geographic location, and the final subgroup was based on the type of fracture.

### 2.5. Statistical analyses

**Figure 1.**
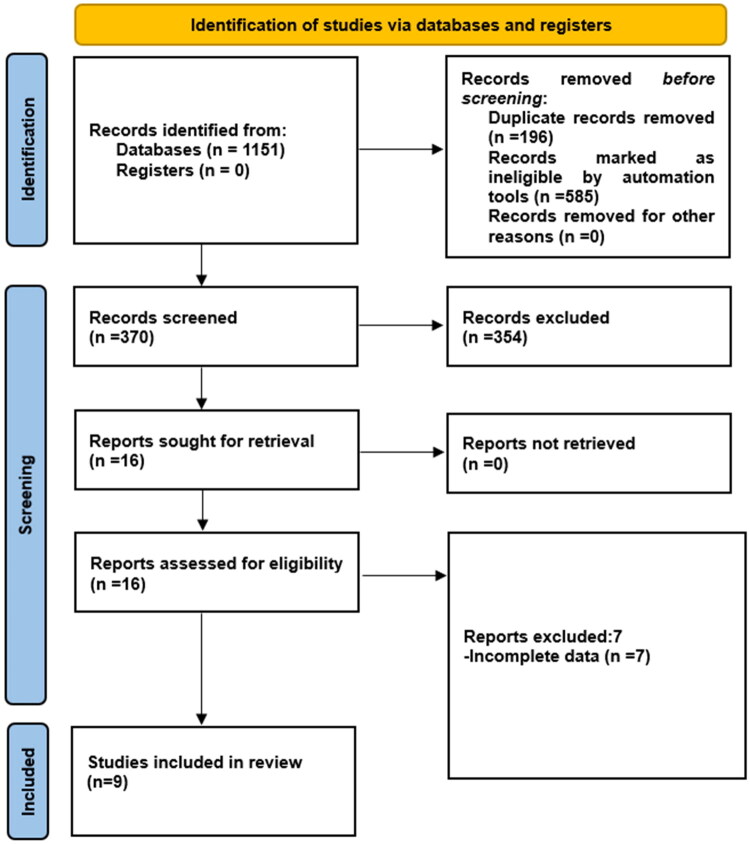
The Preferred Reporting Items for Systematic reviews and Meta-analysis flow diagram to show study selection.

The risk ratio (RR) with 95% confidence intervals (CI) was used as the effect measure for dichotomous outcomes. A two-sided *p* < 0.05 was considered statistically significant. A Random Effects Model (Mantel-Haenszel method) was utilised for all meta-analyses to account for potential between-study heterogeneity. Further, the potential for publication bias was assessed using funnel plots with Egger weighted regression test, when a sufficient number of studies (*n* > 10) were available. Finally, to assess the robustness of summary estimates and to detect if any particular study accounted for a large proportion of heterogeneity, sensitivity analysis was performed by the leave-one-out method [18]. All meta-analyses in the current study were conducted using Review Manager (version 5.4) Duplicates were removed using EndNote X9 software. During the identification phase, automation tools were used to exclude records based on document type and language, as detailed in Figure 1.

## 3. Results

### 3.1. Search results

A total of 1,151 articles were initially retrieved according to the search strategy, 196 of which were excluded after removing duplicates, 354 records were excluded because they were irrelevant to the topic, reviews, animal studies, or case reports based on title and abstract screening and 16 were obtained by reading the titles and abstracts in strict accordance with the inclusion and exclusion criteria. Finally, through reading the full text, 7 more studies were excluded (Supplementary material S3) and 9 studies were included. The literature screening process is shown in Figure 1.

### 3.2. Study characteristics

The studies included in this article are all publications after 2020, with one study published in 2020 [23], and the rest published in 2022 [12,24–30],. The types of studies included in the analysis comprise five retrospective studies [24,25,28–30], and four observational studies [12,23,26,27]. Table 1 summarises the basic characteristics of all the studies included in the analysis.

**Table 1.** Baseline characteristics of the included literature.

Author	Year	Region	Study type	Lock down	Classification of fracture	Age	Evidence level
E.Lopez Gavilanez[1]	2022	Ecuador	Retrospective Studies	Yes	Hip fracture	>60	II
G. Ogliari[2]	2020	UK	observational study	Yes	All kinds of fractures	>50	II
T. Oliveira[3]	2022	Canada	Retrospective Studies	Yes	All kinds of fractures	>50	II
J. C. Ormeño[4]	2022	Chile	Retrospective Studies	No	Hip fracture	>65	II
X. Surís[5]	2022	Spain	observational study	Yes	All kinds of fractures	>50	II
R. Wilk[6]	2022	Poland	observational study	Yes	All kinds of fractures	>50	II
G. Salvio[7]	2022	Italy	observational study	No	Vertebral fracture	>60	II
Paccou J[8]	2021	France	Retrospective study	Yes	Hip fracture	>50	III
Lui DTW[9]	2024	China	Retrospective study	No	All kinds of fractures	>50	III

Five studies were conducted in Europe [12,23,26–28], two studies were conducted in South America [25,30], one study was conducted in North America [24] and one study was conducted in China [29]. Table 1 also focused on whether these regions implemented lockdowns, and the results showed that six studies [23,24,26–28,30] mentioned lockdowns while three studies [12,25,29] did not mention any lockdowns. Among all the studies included, three studies [25,28,30] focused on osteoporotic hip fractures, one study [12] focused on osteoporotic vertebral fractures, and five studies [23,24,26,27,29] focused on all types of fractures associated with osteoporosis. Regarding the age of the study population, six studies limited the age of the participants to 50 years and above [23,24,26–29], two studies limited the age of the participants to 60 years and above [12,30] and one study limited the age of the participants to 65 years and above [25]. The level of evidence for seven studies was level II [12,23–27,30], and the remaining two studies were level III [28,29]. All the cohort studies were of high quality, with NOS scores between 8 and 9. In addition, five studies provided the specific incidence rates of osteoporotic fractures during the COVID-19 pandemic [25–27,29,30]. During the pandemic, Lopez Gavilanez et al. [30] reported an incidence rate of 110 cases of osteoporotic hip fractures per 100,000 people. Wilk et al. [27] reported an incidence rate of 288 cases of osteoporotic hip fractures per 100,000 people. Surís et al. [26] reported an incidence rate of 260 cases of osteoporotic hip fractures per 100,000 people. Cristóbal Ormeño et al. [25] reported an incidence rate of 274 cases of osteoporotic hip fractures per 100,000 people. Lui et al. [29] reported an incidence rate of 271 cases of osteoporotic hip fractures per 100,000 people. Additionally, Wilk et al. [27] reported an incidence rate of 667 cases of all types of osteoporotic fractures per 100,000 people. Surís et al. [26] reported an incidence rate of 848 cases of all types of osteoporotic fractures per 100,000 people. Besides reporting the incidence rate, two studies reported the mortality rate as an outcome measure [12,30], one study reported hospitalization rate ratios as an outcome measure [28] and one study reported complications as an outcome measure [24]. This systematic review also summarized all the conclusions that were included in the literature. Both Lopez Gavilanez et al. [30] and Oliveira et al. [24] concluded that there was a decreased incidence of osteoporotic fractures during the COVID-19 pandemic. The conclusion of Salvio et al. [12]. was that supplementing with vitamin D during the COVID-19 pandemic reduced the incidence of fractures in patients with osteoporosis. The study by Wilk et al. [27] demonstrated a decrease in incidence of osteoporotic fractures during the first few months of the COVID-19 pandemic, which was not statistically significant over a one-year observational period. Lui et al.’s [29] study demonstrated increased risk of major osteoporotic fractures after SARS-CoV-2 infection in both acute and post-acute phases in older adults, partly due to increased fall risk. Ogliari et al. [23] and Surís et al. [26] arrived at a similar conclusion that there was a decreased incidence of non-hip osteoporotic fractures during the COVID-19 pandemic, while the incidence of osteoporotic hip fractures was unchanged compared to before the pandemic. It is interesting to note that Paccou et al. [28] arrived at a completely opposite conclusion, they showed that hospitalizations for hip fractures in France decreased by 11% during the first nationwide COVID-19 lockdown.

### 3.3. Quality evaluation

Supplementary material S4 describes the results of the bias risk assessment for each study included in the final evidence synthesis. All the included studies were of mild to high quality, with NOS scores between 6 and 8. (Supplementary material S4).

### 3.4. Association between the COVID-19 pandemic and the incidence of osteoporotic fractures

**Figure 2.**
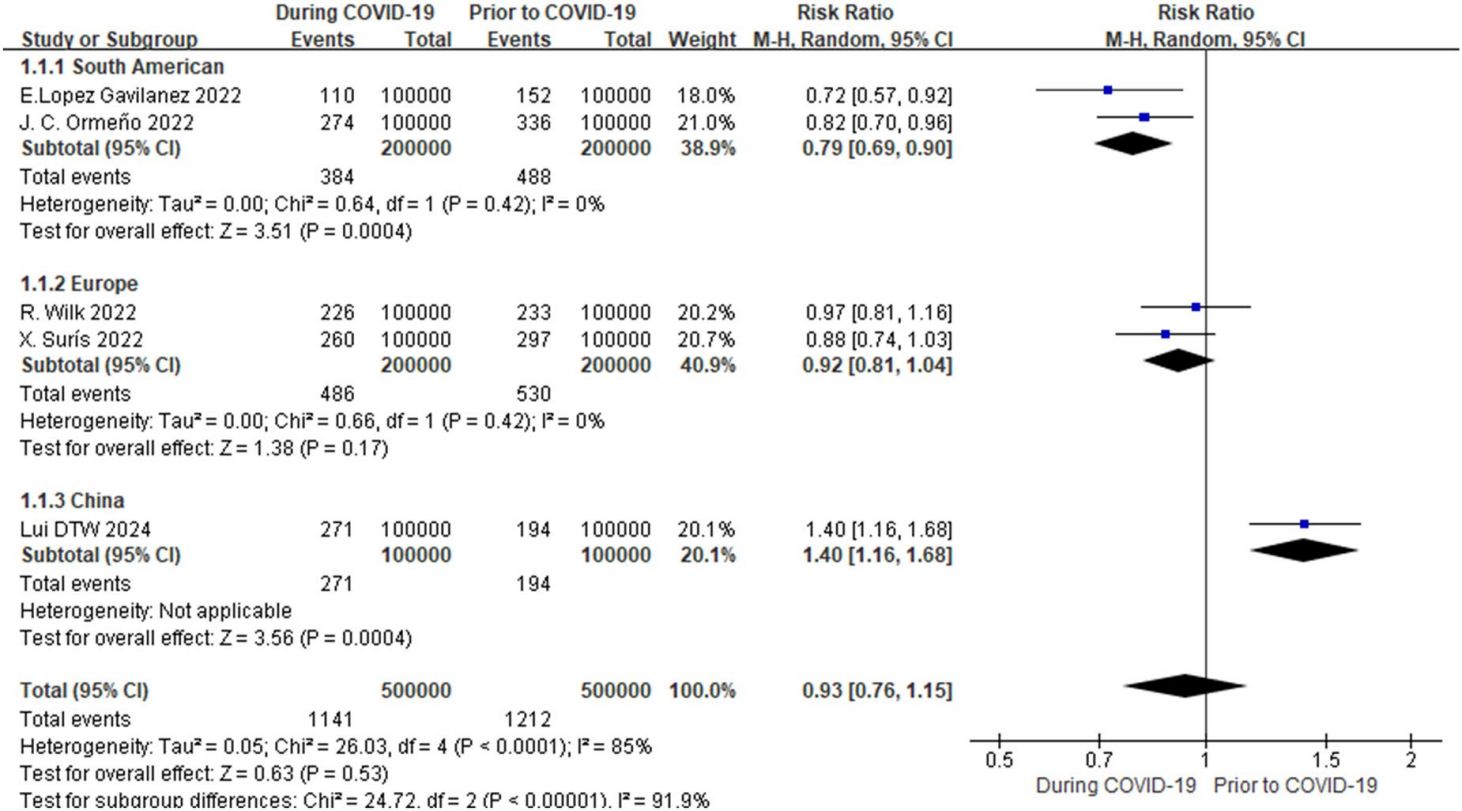
Meta-analysis of the impact of the COVID-19 pandemic on osteoporotic HIP fracture incidence in different geographic regions.

Subgroup analysis by geographic region showed that the incidence of osteoporotic hip fractures during the COVID-19 pandemic in South America was lower than before the pandemic (RR = 0.79, 95% CI 0.69–0.90, *p* = 0.0004, *I^2^*=0%). But the incidence of osteoporotic hip fractures during the COVID-19 pandemic is higher than before the pandemic in Hong Kong, China (RR = 1.40, 95% CI 1.16–1.68, *p* = 0.0004). Our results showed a lack of evidence for a change in the incidence of osteoporotic hip fractures in European populations before and during the COVID-19 pandemic (RR = 0.93, 95% CI 0.76–1.15, *p* = 0.53, *I^2^*=85%) (Figure 2).

**Figure 3.**
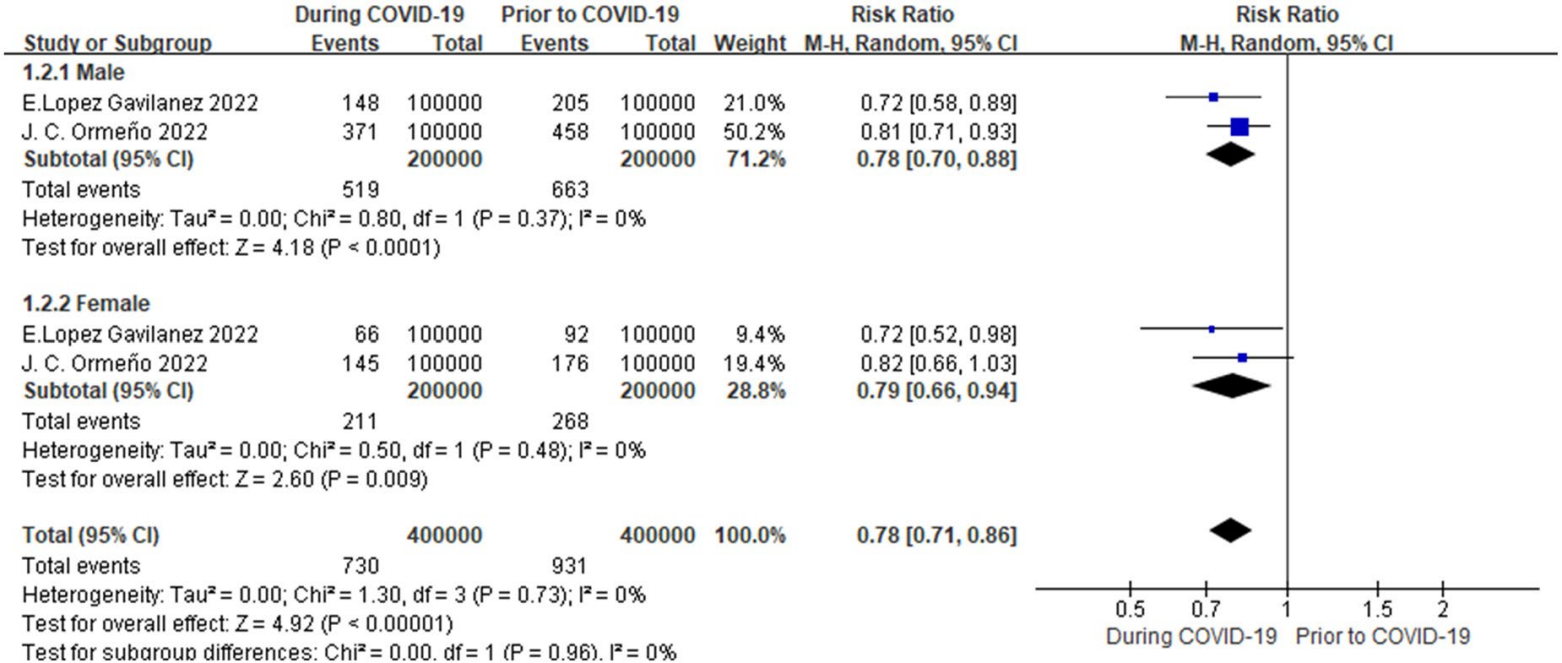
Meta-analysis of the impact of the COVID-19 pandemic on osteoporotic HIP fracture incidence in different sexes.

Subgroup analysis by sex showed that both males (RR = 0.78, 95% CI 0.70–0.88, *p* < 0.0001, I^2^=0%) and females (RR = 0.79, 95% CI 0.66–0.94, *p* = 0.009, *I^2^*=0%) had a lower incidence of osteoporotic hip fractures during the COVID-19 pandemic than before the pandemic (Figure 3).

**Figure 4.**
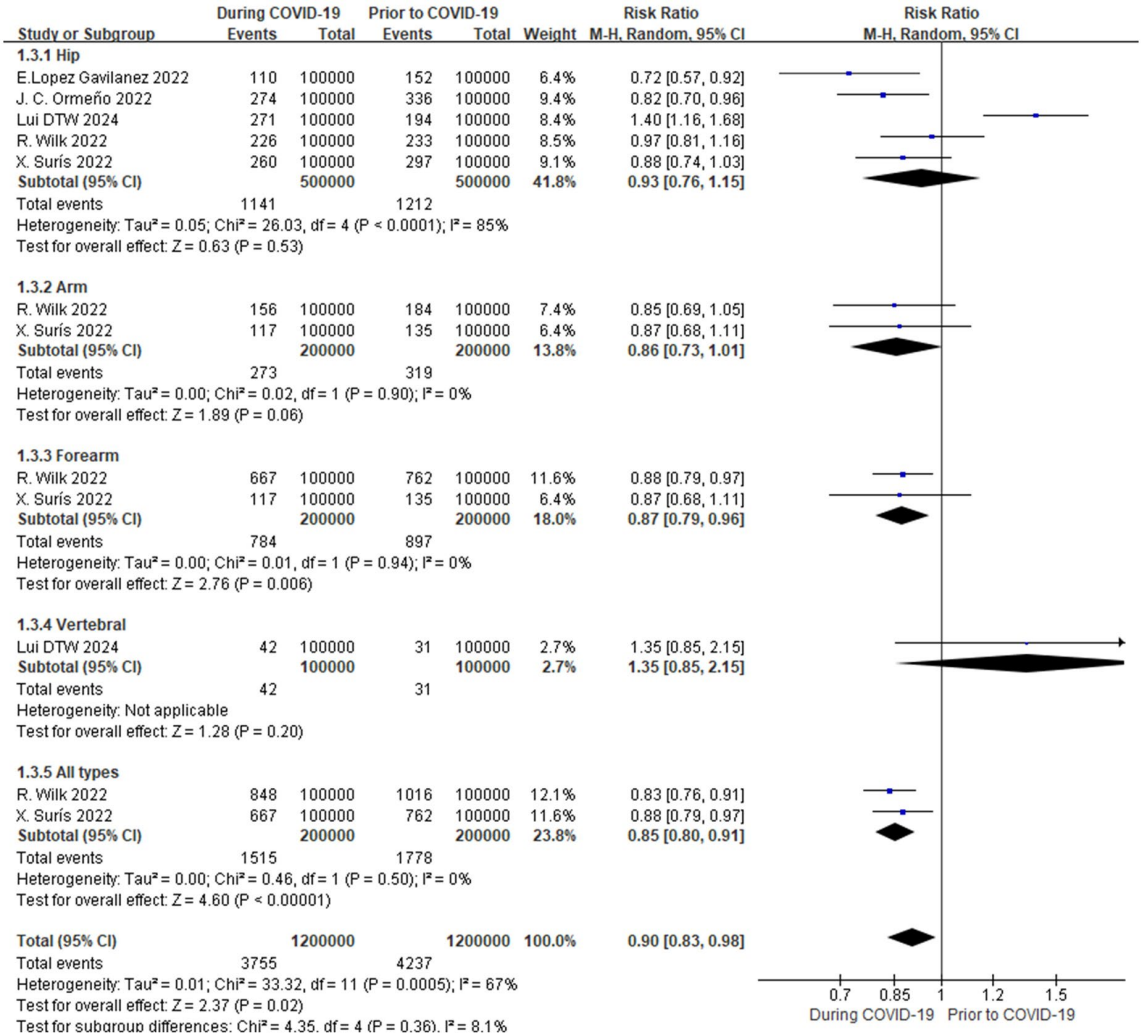
Meta-analysis of the impact of the COVID-19 pandemic on osteoporotic fracture incidence in different types of fracture.

Overall, the pooled analysis of included studies indicated a general downward trend in the incidence of osteoporotic fractures during the pandemic. Specifically, for the ‘all types’ category of osteoporotic fractures, a statistically significant reduction was observed, with a pooled risk ratio (RR) of 0.85 (95% CI 0.80–0.91, *p* < 0.00001) (Figure 4). Based on subgroup analysis by different fracture types (hip, forearm, vertebral, arm and all types), the incidence of osteoporotic forearm fractures (RR = 0.82, 95% CI 0.73–0.93, *p* = 0.0001, *I^2^*=0%), and osteoporotic all types fractures (RR = 0.85, 95% CI 0.80–0.91, *p* < 0.002, *I^2^*=0%) were lower during the COVID-19 pandemic than before the pandemic. Furthermore, our results indicated a lack of evidence for a change in the incidence of osteoporotic arm fractures(RR = 0.86, 95% CI 0.73–1.01, *p* = 0.06, *I^2^*=0%), osteoporotic hip fractures (RR = 0.94, 95% CI 0.87–1.02, *p* = 0.14, *I^2^*=85%), and osteoporotic vertebral fractures(RR = 1.35, 95% CI 0.85–2.15, *p* = 0.20) before and during the COVID-19 pandemic (Figure 4). Subgroup analysis for vertebral fractures included studies with available incidence data. The study by Salvio et al. [12], while included in the systematic review (Table 1), was excluded from the meta-analysis (Figure 4) because it lacked the raw event/population data necessary for calculating risk ratios.

## 4. Discussion

The impact of the COVID-19 pandemic on the healthcare of patients with conditions other than COVID-19 has been studied in various fields, but there is still controversy over the extent and scope of its impact depending on the pathology and geographical region. Regarding osteoporosis, the results of this meta-analysis revealed specific changes in fracture patterns during the COVID-19 pandemic. Our meta-analysis of all included studies indicates an overall 15% reduction in fracture incidence (RR = 0.85). However, it is crucial to note that this “overall decreasing trend” was largely driven by a reduction in minor fractures (such as forearm fractures) and pooled “all types” data. In contrast, the incidence of major osteoporotic fractures—specifically hip and vertebral fractures—did not show a statistically significant change globally. In terms of sex, the incidence of osteoporotic hip fractures showed a decreasing trend in both males and females. However, based on our current evidence, the incidence of osteoporotic hip fractures in Europe did not show a significant change before and after the COVID-19 pandemic.

According to Pluskiewicz et al. [31], the incidence of osteoporotic fractures in the arm, forearm, and hip decreased significantly during the COVID-19 pandemic. The largest decrease in incidence of osteoporotic fractures during the pandemic was observed in the forearm [32]. Similar observations were made in other regions of Poland as well [33]. The possible reasons for the decrease in the incidence of forearm osteoporotic fractures during the pandemic include: Firstly, forearm fractures are most commonly caused by outdoor walking [34], and the restriction of people’s mobility due to the isolation during the COVID-19 pandemic may have led to a decrease in the occurrence of fractures. Secondly, patients may not immediately seek medical attention for fracture diagnosis because there is a possibility of contracting COVID-19 in hospitals. Finally, the existence of the isolation period may lead to a gradual reduction in the patient’s symptoms. Due to the impact of the pandemic, hospitals have faced shortages of staff and resources, which may have affected the treatment and surgical scheduling of fracture patients and led to a decrease in the reported occurrence of fractures. According to the references included in this article [26,27], a meta-analysis found that there was no statistically significant difference in the incidence of osteoporotic arm fractures (referring to humerus fractures in the context) during the COVID-19 pandemic compared to before the pandemic. This suggests that the COVID-19 pandemic did not have a significant impact on the occurrence of osteoporotic arm fractures. This might be because arm fractures tend to occur more frequently during outdoor activities in the winter season [35], and the two studies included in this article were not conducted during the winter season. Therefore, the results of the meta-analysis indicate that there was no significant change in the incidence of osteoporotic arm fractures before and during the COVID-19 pandemic.

Based on the current evidence, the incidence of osteoporotic hip fractures also appears to have decreased during the COVID-19 pandemic. However, the situation with osteoporotic hip fractures is different from forearm fractures, because the pain caused by hip fractures prompts patients to seek medical attention immediately. As a result, any changes in the incidence of hip fractures during the COVID-19 pandemic may be more accurately reflected in the available data compared to forearm fractures. We believe that due to the implementation of isolation and other restrictive measures, entire family groups were able to stay at home, and family members were able to provide more care and attention to their older relatives. As a result, there has been a decrease in the incidence of osteoporotic fractures caused by falls among the older population. Other studies have also shown a decrease in the number of hip fractures during the COVID-19 pandemic in Europe [23,36,37], Asia [38], and Latin America [39–41]. In addition, literature reports have shown a decrease in the average length of hospital stay for hip fracture patients [36].

Indeed, whether there has been a change in the incidence of hip fractures remains a worthwhile question for further investigation. The impact of the COVID-19 pandemic on the number of hip fracture patients varies in different regions. In India, Hong Kong, and Spain, a decrease in the incidence of hip fractures has been observed [32,37,42]. However, one study suggested that SARS-CoV-2 may lead to hypocalcemia, altered bone turnover markers, and a high prevalence of vertebral fractures [43]. Whereas Wilk et al. found that lockdown measures did not affect the overall incidence of hip fractures [44]. Yalamchi et al. [45] found a slight increase in the number of hip fractures per month in Iran during the pandemic, but this was not statistically significant. This could be due to different preventive measures taken by countries regarding COVID-19, or it could be due to differences in ethnicity. However, more high-quality research is needed to further elucidate this point.

A notable discrepancy was observed between regions: while South America showed a significant decrease in hip fractures (RR = 0.79), Lui et al. reported a significant increase in Hong Kong (RR = 1.40). This divergence likely reflects different underlying mechanisms. The South American studies assessed the general population during lockdowns, where restricted outdoor mobility likely reduced trauma-related fractures [25,30]. In contrast, Lui et al. [29] specifically investigated fracture risk following SARS-CoV-2 infection. The increase in Hong Kong likely reflects the biological sequelae of the virus, such as post-infection frailty, neuromuscular weakness, and increased fall risk, rather than environmental lockdown effects.

Although our meta-analysis indicates a general downward trend in fracture incidence, likely due to reduced mobility and trauma during lockdowns, this should not be interpreted as the pandemic being “protective” for bone health. Recent evidence highlights a detrimental association between SARS-CoV-2 and the skeleton. As reviewed by Creecy et al. [43], SARS-CoV-2 infection can induce bone loss through direct mechanisms and indirect factors. Furthermore, the relationship is bidirectional. Pre-existing skeletal fragility negatively impacts COVID-19 outcomes. di Filippo et al. [46] demonstrated that morphometric vertebral fractures at hospitalisation are independent predictors of Long COVID syndrome and impaired respiratory recovery. Therefore, the reduction in fracture presentations observed in our study may also reflect barriers to accessing healthcare rather than a true preservation of bone quality.

This study still has the following limitations, which need to be considered when interpreting the present findings: (1) Without long-term follow-up, the long-term results are not clear. (2) The literature evidence included in this article is all level II, and no randomized controlled trials were included. (3) We only studied populations from some regions, and further research is needed from more regions to increase the credibility of the results. (4) All included studies were based on survivors during the COVID-19 pandemic. We cannot rule out the possibility that some people who died at home may have suffered undetected osteoporotic fractures before death. (5) Only five studies reported the specific incidence of osteoporotic fractures in the population. (6) The current results from the meta-analysis only demonstrate a correlation between osteoporotic fractures and the COVID-19 pandemic, and cannot be used to establish causation. (7) Additionally, due to the lack of stratified data in the primary studies, we were unable to perform a separate subgroup analysis comparing fracture incidence between SARS-CoV-2 infected and non-infected individuals. Consequently, our results reflect the overall epidemiological trend during the pandemic period rather than the direct pathophysiological effect of the virus on bone.

## 5. Conclusion

During the COVID-19 pandemic, there was a decrease in the incidence of minor fractures, such as those of the forearm, likely due to reduced outdoor activity. However, the incidence of major osteoporotic fractures (hip and vertebral) remained stable globally, with significant reductions observed only in specific regions like South America.

## Supplementary Material

Supplementary Material S3.docx

Supplementary Material S4.docx

Supplementary Material S2.docx

Supplementary Material S1.docx

## Data Availability

The data that support the findings of this study are available on request from the corresponding author, upon reasonable request and with the provision of a data sharing agreement.
