# Supplementary material for: The impact of the COVID-19 pandemic on osteoporotic fractures: a systematic review and meta-analysis
Source: Ann Med. 2025 Dec 22;58(1):2604391. doi: 10.1080/07853890.2025.2604391 (PMC12724176; doi:10.1080/07853890.2025.2604391)
Supplement: Supplementary Material S3.docx [file IANN_A_2604391_SM5307.docx]

| Alsadhan, Alyusuf [1] | Incomplete data |
| --- | --- |
| Kang, Wen [2] | Incomplete data |
| McCloskey [3] | Incomplete data |
| Oliveira [4] | Incomplete data |
| Peeters,[5] | Incomplete data |
| Tang [6] | Incomplete data |
| Yu, Tsourdi [7] | Incomplete data |

1. Alsadhan I, Alyusuf EY, Shaltoot O, AlRuwashid S, Alhamad M, Ekhzaimy A, et al. (2023) Adoption of telemedicine care for osteoporotic patients during the COVID-19 pandemic: experience from a tertiary care center in Saudi Arabia. Archives of Osteoporosis 18:

2. Kang X, Wen X, Liang J, Liu L, Zhang Y, Wang Q, et al. (2022) The Biological Interaction of SARS-CoV-2 Infection and Osteoporosis: A Preliminary Study. Frontiers in Cell and Developmental Biology 10:

3. McCloskey EV, Harvey NC, Johansson H, Lorentzon M, Vandenput L, Liu E, et al. (2021) Global impact of COVID-19 on non-communicable disease management: descriptive analysis of access to FRAX fracture risk online tool for prevention of osteoporotic fractures. Osteoporosis International 32:39-46

4. Oliveira T, Brown J, Juby AG, Schneider P, Wani RJ, Packalen M, et al. (2022) Trends in osteoporosis care patterns during the COVID-19 pandemic in Alberta, Canada. Archives of Osteoporosis 17:

5. Peeters JJM, van den Berg P, van den Bergh JP, Emmelot-Vonk MH, de Klerk G, Lems WF, et al. (2021) Osteoporosis care during the COVID-19 pandemic in the Netherlands: A national survey. Archives of Osteoporosis 16:

6. Tang J (2022) COVID-19 Pandemic and Osteoporosis in Elderly Patients. Aging and Disease 13:960-969

7. Yu EW, Tsourdi E, Clarke BL, Bauer DC, Drake MT (2020) Osteoporosis Management in the Era of COVID-19. Journal of Bone and Mineral Research 35:1009-1013
