# Supplementary material for: The impact of the COVID-19 pandemic on osteoporotic fractures: a systematic review and meta-analysis
Source: Ann Med. 2025 Dec 22;58(1):2604391. doi: 10.1080/07853890.2025.2604391 (PMC12724176; doi:10.1080/07853890.2025.2604391)
Supplement: Supplementary Material S4.docx [file IANN_A_2604391_SM5306.docx]

**Supplementary Material S4:** Quality assessment and publication bias evaluation of included study.

**Table 1:** The cohort study was based on the NOS quality assessment form.

| **Author** | **Representativeness of exposed cohort** | **Selection** | **Ascertainment of exposure** | **Demonstration that outcome of interest was not present at the start of study** | **Study control for IBLA** | **Study control for Traditional surgery** | **Assessment of outcome** | **Was follow-up long enough for outcomes to occur** | **Adequacy of follow up of cohorts** |
| --- | --- | --- | --- | --- | --- | --- | --- | --- | --- |
| Lopez Gavilanez [1] | √ | √ | √ | √ | √ | √ | √ | √ | √ |
| Wilk [2] | √ | √ | √ | √ | √ | √ | √ | √ | √ |
| Surís [3] | √ | √ | √ | √ | √ | √ | √ | √ | √ |
| Salvio [4] | √ | √ | √ | √ | √ | √ | √ | √ | √ |
| Ormeño [5] | √ | √ | √ | √ | √ | √ | √ | √ | √ |
| Oliveira [6] | √ | √ | √ | √ | √ | √ | √ | √ | √ |
| Ogliari [7] | √ | √ | √ | √ | √ | √ | √ | √ | √ |
| Paccou J[8] | √ | √ | √ | √ | √ | √ | √ | √ | √ |
| Lui DTW[9] | √ | √ | √ | √ | √ | √ | √ | √ | √ |

1. Lopez Gavilanez, E., et al., *Decreasing incidence rates of osteoporotic hip fractures in Ecuador during the COVID-19 pandemic.* Archives of Osteoporosis, 2023. **18**(1).

2. Wilk, R., et al., *One year of the COVID-19 pandemic in Poland–the incidence of osteoporotic forearm, arm, and hip fractures.* Archives of Osteoporosis, 2022. **17**(1).

3. Surís, X., et al., *Effects of COVID-19 confinement on the incidence and mortality of major osteoporotic fractures: an observational study in Catalonia, Spain.* Archives of Osteoporosis, 2022. **17**(1).

4. Salvio, G., et al., *Remote management of osteoporosis in the first wave of the COVID-19 pandemic.* Archives of Osteoporosis, 2022. **17**(1).

5. Ormeño, J.C., et al., *Impact of the COVID-19 pandemic on osteoporotic hip fractures in Chile.* Archives of Osteoporosis, 2022. **17**(1).

6. Oliveira, T., et al., *Trends in osteoporotic fracture and related in-hospital complications during the COVID-19 pandemic in Alberta, Canada.* Archives of Osteoporosis, 2022. **17**(1).

7. Ogliari, G., et al., *The impact of lockdown during the COVID-19 pandemic on osteoporotic fragility fractures: an observational study.* Archives of Osteoporosis, 2020. **15**(1).

8. Paccou, J., et al., *Analysis of Hip Fractures in France During the First COVID-19 Lockdown in Spring 2020.* JAMA Netw Open, 2021. **4**(11): p. e2134972.

9. Lui, D.T.W., et al., *Risks of incident major osteoporotic fractures following SARS-CoV-2 infection among older individuals: a population-based cohort study in Hong Kong.* J Bone Miner Res, 2024. **39**(5): p. 551-560.
