# Supplementary material for: The impact of the COVID-19 pandemic on osteoporotic fractures: a systematic review and meta-analysis
Source: Ann Med. 2025 Dec 22;58(1):2604391. doi: 10.1080/07853890.2025.2604391 (PMC12724176; doi:10.1080/07853890.2025.2604391)
Supplement: Supplementary Material S2.docx [file IANN_A_2604391_SM5305.docx]

# ****Web of science****

**(((((((((((((((((((((TS=(Osteoporosis)) OR TS=(Osteoporoses)) OR TS=(Osteoporosis, Post-Traumatic)) OR TS=(Osteoporosis, Post Traumatic)) OR TS=(Post-Traumatic Osteoporoses)) OR TS=(Post-Traumatic Osteoporosis)) OR TS=(Osteoporosis, Senile)) OR TS=(Osteoporoses, Senile)) OR TS=(Senile Osteoporoses)) OR TS=(Osteoporosis, Involutional)) OR TS=(Senile Osteoporosis)) OR TS=(Osteoporosis, Age-Related)) OR TS=(Osteoporosis, Age Related)) OR TS=(Bone Loss, Age-Related)) OR TS=(Age-Related Bone Loss)) OR TS=(Age-Related Bone Losses)) OR TS=(Bone Loss, Age Related)) OR TS=(Bone Losses, Age-Related)) OR TS=(Age-Related Osteoporosis)) OR TS=(Age Related Osteoporosis)) OR TS=(Age-Related Osteoporoses)) OR TS=(Osteoporoses, Age-Related)** and **Preprint Citation Index** (Exclude – Database) 2010230

**((((((((((((((((((((((((TS=(SARS-CoV-2)) OR TS=(SARS-CoV-2 Virus)) OR TS=(SARS CoV 2 Virus)) OR TS=(SARS-CoV-2 Viruses)) OR TS=(Virus, SARS-CoV-2)) OR TS=(2019 Novel Coronavirus)) OR TS=(2019 Novel Coronaviruses)) OR TS=(Coronavirus, 2019 Novel)) OR TS=(Novel Coronavirus, 2019)) OR TS=(COVID-19 Virus)) OR TS=(COVID 19 Virus)) OR TS=(COVID-19 Viruses)) OR TS=(Virus, COVID-19)) OR TS=(Wuhan Coronavirus)) OR TS=(Coronavirus, Wuhan)) OR TS=(COVID19 Virus)) OR TS=(COVID19 Viruses)) OR TS=(Virus, COVID19)) OR TS=(Viruses, COVID19)) OR TS=(Coronavirus Disease 2019 Virus)) OR TS=(Severe Acute Respiratory Syndrome Coronavirus 2)) OR TS=(SARS Coronavirus 2)) OR TS=(Coronavirus 2, SARS)) OR TS=(2019-nCoV)) OR TS=(Wuhan Seafood Market Pneumonia Virus)** and **Preprint Citation Index** (Exclude – Database)

299272

**#124 AND #116** and **Preprint Citation Index** (Exclude – Database) 248

# Embase


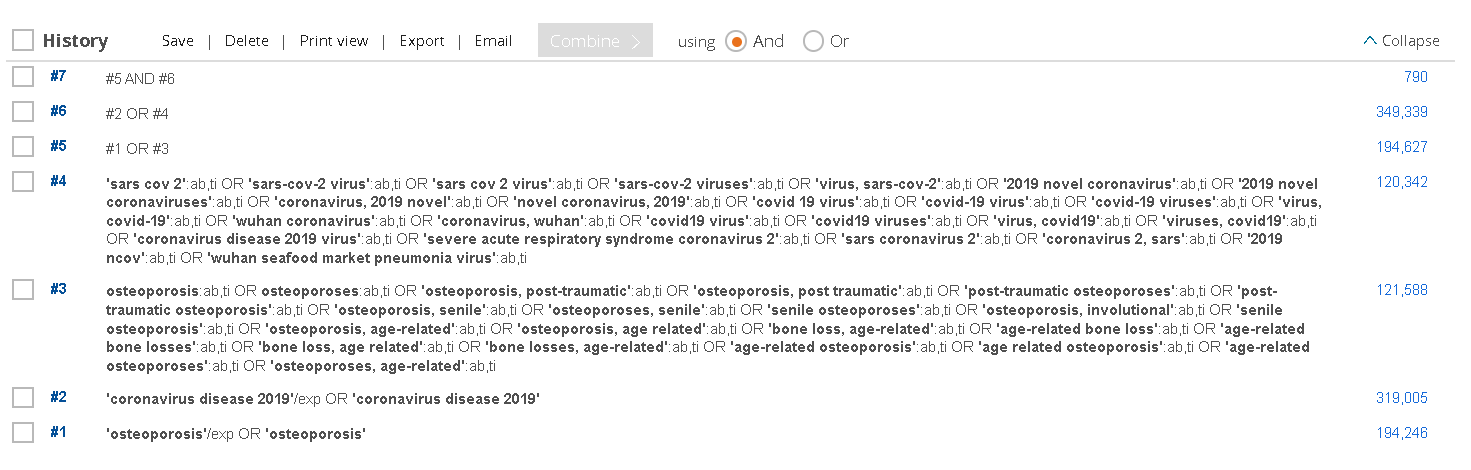


# Cochrane

Search Name:

Date Run: 18/04/2023 22:45:41

Comment:

ID Search Hits

#1 MeSH descriptor: [Osteoporosis] explode all trees 5754

#2 MeSH descriptor: [COVID-19] explode all trees 4093

#3 (Osteoporoses):ti,ab,kw OR (Osteoporosis, Post-Traumatic):ti,ab,kw OR (Osteoporosis, Post Traumatic):ti,ab,kw OR (Post-Traumatic Osteoporoses):ti,ab,kw OR (Post-Traumatic Osteoporosis):ti,ab,kw 25

#4 (Osteoporosis, Senile):ti,ab,kw OR (Osteoporoses, Senile):ti,ab,kw OR (Senile Osteoporoses):ti,ab,kw OR (Osteoporosis, Involutional):ti,ab,kw OR (Senile Osteoporosis):ti,ab,kw 152

#5 (Osteoporosis, Age-Related):ti,ab,kw OR (Osteoporosis, Age Related):ti,ab,kw OR (Bone Loss, Age-Related):ti,ab,kw OR (Age-Related Bone Loss):ti,ab,kw OR (Age-Related Bone Losses):ti,ab,kw 799

#6 (Bone Loss, Age Related):ti,ab,kw OR (Bone Losses, Age-Related):ti,ab,kw OR (Age-Related Osteoporosis):ti,ab,kw OR (Age Related Osteoporosis):ti,ab,kw OR (Age-Related Osteoporoses):ti,ab,kw 1119

#7 (Osteoporoses, Age-Related):ti,ab,kw 0

#8 (SARS-CoV-2):ti,ab,kw OR (SARS-CoV-2 Virus):ti,ab,kw OR (SARS CoV 2 Virus):ti,ab,kw OR (SARS-CoV-2 Viruses):ti,ab,kw OR (Virus, SARS-CoV-2):ti,ab,kw 580

#9 (2019 Novel Coronavirus):ti,ab,kw OR (2019 Novel Coronaviruses):ti,ab,kw OR (Coronavirus, 2019 Novel):ti,ab,kw OR (Novel Coronavirus, 2019):ti,ab,kw OR (COVID-19 Virus):ti,ab,kw 3344

#10 (COVID 19 Virus):ti,ab,kw OR (COVID-19 Viruses):ti,ab,kw OR (Virus, COVID-19):ti,ab,kw OR (Wuhan Coronavirus):ti,ab,kw OR (Coronavirus, Wuhan):ti,ab,kw 3021

#11 (COVID19 Virus):ti,ab,kw OR (COVID19 Viruses):ti,ab,kw OR (Virus, COVID19):ti,ab,kw OR (Viruses, COVID19):ti,ab,kw OR (Coronavirus Disease 2019 Virus):ti,ab,kw 1272

#12 (Severe Acute Respiratory Syndrome Coronavirus 2):ti,ab,kw OR (SARS Coronavirus 2):ti,ab,kw OR (Coronavirus 2, SARS):ti,ab,kw 4145

#13 #1 OR #3 OR #4 OR #5 OR #6 OR #7 6652

#14 #2 OR #8 OR #9 OR #10 OR #11 OR #12 8394

#15 #13 AND #14 4

# Pubmed

(("Osteoporosis"[Mesh]) OR ((((((((((((((((((((((Osteoporosis[Title/Abstract]) OR (Osteoporoses[Title/Abstract])) OR (Osteoporosis, Post-Traumatic[Title/Abstract])) OR (Osteoporosis, Post Traumatic[Title/Abstract])) OR (Post-Traumatic Osteoporoses[Title/Abstract])) OR (Post-Traumatic Osteoporosis[Title/Abstract])) OR (Osteoporosis, Senile[Title/Abstract])) OR (Osteoporoses, Senile[Title/Abstract])) OR (Senile Osteoporoses[Title/Abstract])) OR (Osteoporosis, Involutional[Title/Abstract])) OR (Senile Osteoporosis[Title/Abstract])) OR (Osteoporosis, Age-Related[Title/Abstract])) OR (Osteoporosis, Age Related[Title/Abstract])) OR (Bone Loss, Age-Related[Title/Abstract])) OR (Age-Related Bone Loss[Title/Abstract])) OR (Age-Related Bone Losses[Title/Abstract])) OR (Bone Loss, Age Related[Title/Abstract])) OR (Bone Losses, Age-Related[Title/Abstract])) OR (Age-Related Osteoporosis[Title/Abstract])) OR (Age Related Osteoporosis[Title/Abstract])) OR (Age-Related Osteoporoses[Title/Abstract])) OR (Osteoporoses, Age-Related[Title/Abstract]))) AND ((SARS-CoV-2[MeSH Terms]) OR (((((((((((((((((((((((((SARS-CoV-2[Title/Abstract]) OR (SARS-CoV-2 Virus[Title/Abstract])) OR (SARS CoV 2 Virus[Title/Abstract])) OR (SARS-CoV-2 Viruses[Title/Abstract])) OR (Virus, SARS-CoV-2[Title/Abstract])) OR (2019 Novel Coronavirus[Title/Abstract])) OR (2019 Novel Coronaviruses[Title/Abstract])) OR (Coronavirus, 2019 Novel[Title/Abstract])) OR (Novel Coronavirus, 2019[Title/Abstract])) OR (COVID-19 Virus[Title/Abstract])) OR (COVID 19 Virus[Title/Abstract])) OR (COVID-19 Viruses[Title/Abstract])) OR (Virus, COVID-19[Title/Abstract])) OR (Wuhan Coronavirus[Title/Abstract])) OR (Coronavirus, Wuhan[Title/Abstract])) OR (COVID19 Virus[Title/Abstract])) OR (COVID19 Viruses[Title/Abstract])) OR (Virus, COVID19[Title/Abstract])) OR (Viruses, COVID19[Title/Abstract])) OR (Coronavirus Disease 2019 Virus[Title/Abstract])) OR (Severe Acute Respiratory Syndrome Coronavirus 2[Title/Abstract])) OR (SARS Coronavirus 2[Title/Abstract])) OR (Coronavirus 2, SARS[Title/Abstract])) OR (2019-nCoV[Title/Abstract])) OR (Wuhan Seafood Market Pneumonia Virus[Title/Abstract]))) 109
